# Investigation into the health effects of reduced chymase function using predicted loss-of-function mutations in *CMA1*

**DOI:** 10.1007/s12265-022-10261-w

**Published:** 2022-05-05

**Authors:** Zammy Fairhurst-Hunter, Robin G. Walters, Alexander Zink, Kuang Lin, Yu Guo, Canqing Yu, Jun Lv, Liming Li, Daniel F. Freitag, Zhengming Chen, Iona Y. Millwood

**Affiliations:** 1 Clinical Trial Service Unit & Epidemiological Studies Unit (CTSU), Nuffield Department of Population Health, Big Data Institute Building, Roosevelt Drive, University of Oxford, UK; 2 Medical Research Council Population Health Research Unit (MRC PHRU) at the University of Oxford, Nuffield Department of Population Health, University of Oxford, UK; 3 Bayer AG, Research and Development, Pharmaceuticals, Biomedical Data Science II (Wup/Ber), Wuppertal, Germany; 4 Chinese Academy of Medical Sciences, Building C, NCCD, Shilongxi Road, Beijing, China; 5 Department of Epidemiology and Biostatistics, School of Public Health, Peking University, 38 Xueyuan Road, Beijing, China

**Keywords:** CMA1, Chymase, pLoF variants, China, CVD, Heart failure, Fibrosis, Chronic Kidney disease

## Abstract

Tissue remodelling and fibrosis which occur in response to injury play a central role in the development of many diseases. Chymase is a key enzyme believed to mediate these pathological processes. As such chymase inhibitors have been under active development for the treatment of a number of conditions. To investigate the impact of reduced chymase function, we constructed a genetic score from two pLoF mutations in the gene encoding chymase and tested its association with diseases and biomarkers. Our study found no association between the genetically-predicted reduced chymase function score and heart failure, chronic kidney disease or other predefined conditions. We additionally found no association of the score with any physical measurements or biomarkers. Our results provide no evidence in support of chymase inhibition as a novel therapeutic strategy for the treatment or prevention of heart failure, chronic kidney disease or major cardiovascular events, as previously proposed.

Chymase is a serine protease secreted from mast cells in response to tissue injury and inflammation. It is encoded in humans by the *CMA1* gene. The enzyme contributes, via its role in conversion of Angiotensin I to Angiotensin II and activation of the proteins MMP-9 and TGF-β, towards the processes of fibrosis and tissue remodelling which underlie the pathology of numerous diseases[1]. Thus, chymase inhibition may represent a novel treatment for various chronic conditions.

Animal studies showed that cardiac fibrosis and renal dysfunction can be reduced by chymase inhibition, leading to the hypothesis that chymase inhibitors may be beneficial for treatment of heart failure (HF) and chronic kidney disease (CKD)[1, 2]. However, the relevance of these results to humans is unclear, particularly as the model animals studied express multiple chymase isoforms[2]. Phase 1 clinical trials have provided evidence on the safety but not efficacy of chymase inhibitors[1]. To inform ongoing and future clinical development programmes, we used predicted loss-of-function (pLoF) mutations within *CMA1* to assess the potential clinical utility and safety of chymase inhibition for the treatment/prevention of HF, CKD, and other conditions, in a large prospective biobank study.

We identified two variants in *CMA1*, rs150310098 and rs13306254, that were high confidence pLoF mutations (https://gnomad.broadinstitute.org). It is predicted that rs150310098 causes loss of a splice donor site while rs13306254 introduces an early stop codon, with both predicted to result in reduced levels of functional chymase. The two variants are present at appreciable frequencies (~1% minor allele frequency [MAF]) in East Asians but are rare (MAF<1x10^-4^) in other ancestries. To investigate the impact of these pLoF variants, we used data on up to 100,578 genotyped participants from the China Kadoorie Biobank (CKB)[3] of adults from ten areas of China, with follow-up via linkage to disease and death registries and hospital admission records. The variants are present on different haplotypes (LD R^2^=1.2x10^-4^), and were combined additively into an unweighted score for genetically-predicted reduced chymase function (RCF). Using linear and logistic regression models, we associated the genetic score with a range of physical measurements, biomarkers and disease endpoints. Primary endpoints were selected based on the main indications in clinical development programmes. Secondary endpoints were selected to assess potential adverse effects or alternative indications for chymase inhibition.

In CKB, rs150310098 and rs13306254 had an overall MAF of 0.013 (range by 10 study areas 0.009-0.016) and 0.017 (0.010-0.023), respectively. There was no significant association between genetically-predicted RCF and physical measurements or biomarkers (P<0.002; Bonferroni adjusted threshold) (Figure 1A). Of note, the RCE score had no association with three quantitative measures of kidney function; cystatin C, creatinine and eGFR (N ~17,000). In ~5,500 participants with creatinine measured using NMR, there was also no association between the score and creatinine levels (Beta=-0.01 SD, 95%CI (−0.10, 0.08), P=0.83). Genetically-predicted RCF had no association with blood pressure or heart rate (Figure 1A). This is in line with findings from the phase 1 clinical trial, which found chymase inhibitors had no impact on these traits despite the role of angiotensin II in modulation of blood pressure[1]. We also assessed associations of the score with 12 ECG variables, 225 metabolites (Nightingale NMR platform) and 92 proteins (OLINK immuneoncology panel) measured in smaller subsets of participants but found no significant associations (data not shown).

Genetically-predicted RCF was not significantly associated with the pre-defined primary study endpoints: HF, CKD or major cardiovascular events (Figure 1B). Similarly, there were no associations of the score with the secondary disease endpoints (Figure 1B). Notably we found no association with asthma or atrial fibrillation, conditions suggested to be affected by the downstream consequences of chymase’s enzymatic activity and previously reported to show associations with non-functional variants within *CMA1*[4, 5].

The low MAF of the variants and low case numbers for HF and CKD meant our study had ~80% power to detect an effect size of 1.8 for these conditions, but was underpowered to reject the null hypothesis for smaller effect sizes. To further investigate the impact of RCF, we combined the effect sizes of the pLOF variants from GWAS summary statistics for HF and nephrotic syndrome in Biobank Japan (http://jenger.riken.jp/en/result) into a single effect size for the RCF score using fixed-effects meta-analysis. Neither condition showed a significant association with the genetically-predicted RCF score in Biobank Japan (HF: total cases=9,413, OR=0.96, 95% CI (0.84-1.09), P=0.54; Nephrotic syndrome: total cases=957, OR=0.82, 95% CI (0.55-1.22), P=0.33).

These results provide no evidence in support of chymase inhibition as a novel therapeutic option for the treatment or prevention of HF, CKD or major cardiovascular events, as previously proposed[1]. Given that the variants studied were rare in Europeans, this work highlights the importance of large, ancestrally diverse cohorts which widen the available pool of pLoF variants, and supports the use of genetic approaches to provide insights into drug target development.

## Figures and Tables

**Figure 1:**
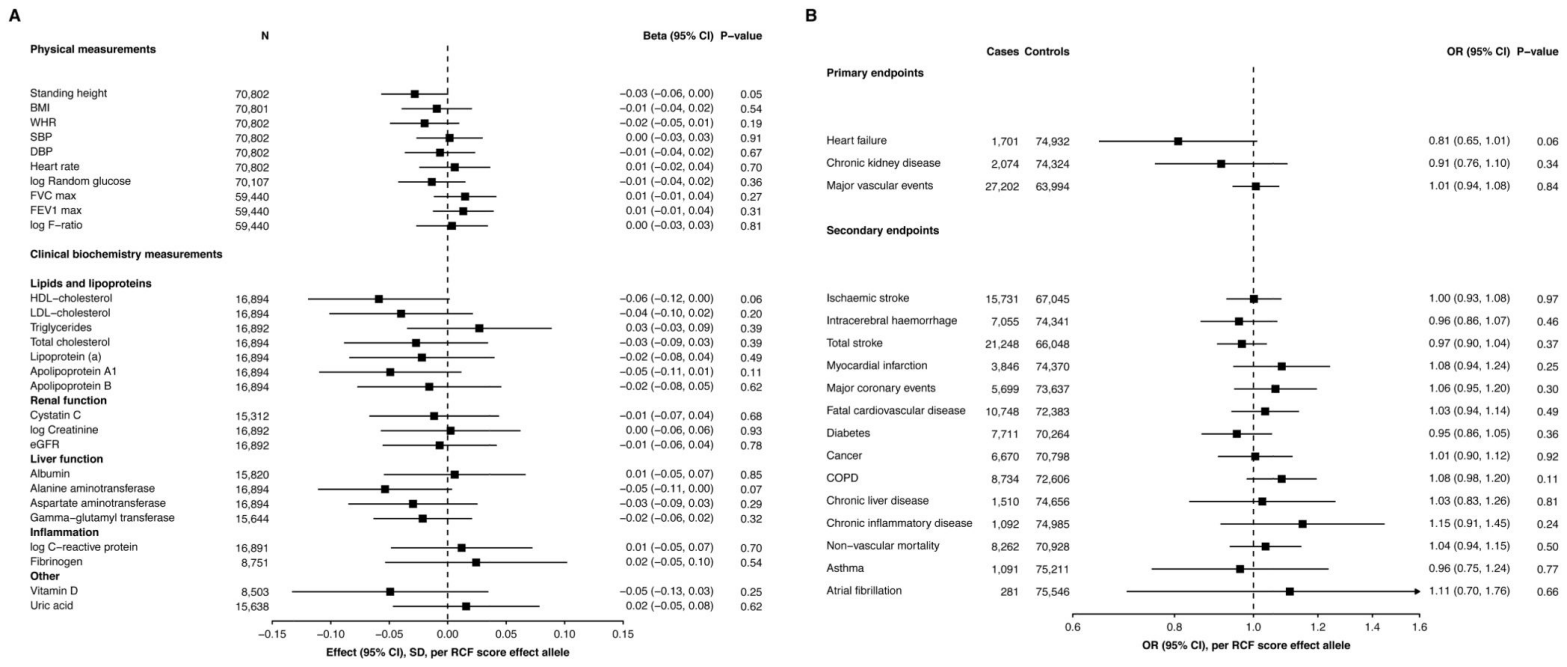
Association of a reduced chymase function (RCF) genetic score with physical measures, biomarkers and disease endpoints A) Results were estimated from the linear regression of each physical measure/biomarker against the genetically-predicted RCF score, including adjustment for age, age^2^, sex, case ascertainment where appropriate and regional principal components. The analysis was stratified by study region and an inverse variance fixed-effects model was used to meta-analyse the effect estimates. Threshold for significance after Bonferroni correction for 28 tests, ∝ = 0.002. B) Results estimated from the logistic regression of each endpoint against the genetically-predicted RCF score, including adjustment for age, age^2^, sex, study region and 12 national principal components. Threshold for significance after Bonferroni correction for 17 tests, ∝ = 0.003.

## Non-standard Abbreviations and Acronyms

HF: Heart failure
CKD: Chronic kidney disease
pLoF: Predicted loss-of-function
MAF: Minor allele frequency
CKB: China Kadoorie Biobank
RCF: Reduced chymase function
